# Dealing with missing data in the Center for Epidemiologic Studies Depression self-report scale: a study based on the French E3N cohort

**DOI:** 10.1186/1471-2288-13-28

**Published:** 2013-02-21

**Authors:** Noémie Resseguier, Hélène Verdoux, Roch Giorgi, Françoise Clavel-Chapelon, Xavier Paoletti

**Affiliations:** 1 Institut Curie, Biostatistics Department, Paris, France; 2 Inserm, UMR 912, Aix-Marseille Univ, UMR 912, SESSTIM, Marseille, France; 3 University Victor Segalen Bordeaux 2, Bordeaux, France; 4 Inserm U657, Bordeaux, France; 5 Centre for Research in Epidemiology and Population Health, UMRS 1018, Team 9, Institut Gustave Roussy, Villejuif, France; 6 Inserm U900 Institut Curie, Paris, France

**Keywords:** CES-D, Cohort, Missing data, Multiple imputation, Non ignorable, Sensitivity analysis

## Abstract

**Background:**

The Center for Epidemiologic Studies - Depression scale (CES-D) is a validated tool commonly used to screen depressive symptoms. As with any self-administered questionnaire, missing data are frequently observed and can strongly bias any inference. The objective of this study was to investigate the best approach for handling missing data in the CES-D scale.

**Methods:**

Among the 71,412 women from the French E3N prospective cohort (Etude Epidémiologique auprès des femmes de la Mutuelle Générale de l’Education Nationale) who returned the questionnaire comprising the CES-D scale in 2005, 45% had missing values in the scale. The reasons for failure to complete certain items were investigated by semi-directive interviews on a random sample of 204 participants. The prevalence of high depressive symptoms (score ≥16, hDS) was estimated after applying various methods for ignorable missing data including multiple imputation using imputation models with CES-D items with or without covariates. The accuracy of imputation models was investigated. Various scenarios of nonignorable missing data mechanisms were investigated by a sensitivity analysis based on the mixture modelling approach.

**Results:**

The interviews showed that participants were not reluctant to answer the CES-D scale. Possible reasons for nonresponse were identified. The prevalence of hDS among complete responders was 26.1%. After multiple imputation, the prevalence was 28.6%, 29.8% and 31.7% for women presenting up to 4, 10 and 20 missing values, respectively. The estimates were robust to the various imputation models investigated and to the scenarios of nonignorable missing data.

**Conclusions:**

The CES-D scale can easily be used in large cohorts even in the presence of missing data. Based on the results from both a qualitative study and a sensitivity analysis under various scenarios of missing data mechanism in a population of women, missing data mechanism does not appear to be nonignorable and estimates are robust to departures from ignorability. Multiple imputation is recommended to reliably handle missing data in the CES-D scale.

## Background

The Center for Epidemiologic Studies Depression Scale (CES-D Scale) was developed for epidemiologic population-based studies to measure the current level of depressive symptoms (DS) [1]. Frequencies of various feelings during the previous week are self-reported on a four-point scale. The CES-D scale consists of 20 items selected from previously developed scales; 16 items are negatively worded, whereas four items are positively worded. Responses to items of the CES-D scale are summed, each item being scored 0/1/2/3; in most studies, a score equal to or greater than 16 is used as cut-off to indicate high DS (hDS) [1]. As described in the original publication, four factors were identified by exploratory factor analysis [1]. Validation studies have shown that the CES-D was internally consistent, moderately stable over several weeks [1] and months [2,3], and strongly correlated with other measures of clinical depression or DS [1-4]. Due to its simplicity, the CES-D scale can be easily administered as a self-report questionnaire. In particular, it can be easily used in large cohort studies. However, the presence of incomplete observations is a major issue that can create biased estimates or spurious associations. Missing values (MVs) in the CES-D scale are often passed over in the medical literature. Observations of patients with more than four MVs are commonly excluded, even though this cut-off of four is not based on any statistical criterion, while observations with less than four MVs are imputed to the person-mean, even when there is a large proportion of incomplete responders [5]. These types of analyses on complete cases or after single imputation have been repeatedly proved to be biased [6].

Although there is an abundance of statistical literature about missing data, reports of epidemiologic studies dealing with missing data in the context of self-rated psychopathological symptoms are rare [7]. Missing data are usually classified as ignorable including missing completely at random (MCAR) and missing at random (MAR) data, and nonignorable, *ie*, missing not at random (MNAR) data [8]. Ignorable or nonignorable missing data mechanism cannot be identified from the data collected, or only in specific contexts under extra assumptions [9]. Only external data or qualitative studies can help to formulate hypotheses concerning the nonresponse mechanism. Analyses differ considerably according to the expected type of missing data. Multiple imputation, a relatively flexible and general purpose approach to dealing with missing data when the missing data process is ignorable, is now available in standard statistical software [8]. It is based on an imputation model that relates the value to impute to a set of predictors. Predictors of DS have been extensively studied, in order to adapt public health policies [10-12] and can be used for the imputation model. The MNAR hypothesis is rarely investigated, although it has been recommended to perform sensitivity analyses under different models for the nonresponse mechanism [8], *i.e.* by studying how a variation in the imputation model modifies the overall results [13]. Two approaches have been described to impute nonignorable nonresponse: mixture modeling and selection modeling. The mixture modeling approach, by mixing different distributions according to the missing / non-missing status, is attractive and the present study will focus on this approach.

The main objectives of the present study conducted on data from a large prospective cohort were:

• To examine qualitatively the hypotheses concerning the mechanism of MVs in semi-directed interviews of subjects;

• To evaluate the predictive ability of the imputation model only including items of the CES-D scale on a cohort with simulated missing data;

• To evaluate the results of multiple imputation of either the score or of the status regarding depression symptoms (score >16) or multiple imputation of each item with two different imputation models including or not variables other than the CES-D items; complete case analysis and simple imputation of the overall CES-D score to the person-mean were used as comparators;

• To explore the possible biases due to MNAR data

## Methods

### *Study design and inclusion criteria*

This study was performed on the French E3N prospective cohort [14] (Etude Epidémiologique auprès des femmes de la Mutuelle Générale de l’Education Nationale). In 1990, the E3N cohort included 98,995 women born between 1925 and 1950 and covered by a national health plan, covering mostly teachers. Participants were asked to complete self-administered questionnaires every two years addressing medical history, history of hormonal phases, and a variety of lifestyle characteristics. Informed consent was obtained from each participant at the beginning of the study after thorough explanation of the objectives of this study. The study was approved by the French National Commission for Personal Data Protection.

Each questionnaire collected data about depression and psychological disorders, and three of them collected data about psychotropic drug use. The eighth questionnaire, administered in 2005, included the CES-D scale; all women who returned this questionnaire were included in the present study, whether or not they filled in the CES-D scale part. All questionnaires are available on the web [14].

### *Data collection*

#### Variables of interest

The two main variables of interest were the total CES-D score (sum of the 20 items) and a dichotomized hDS variable using the cut-off of 16 [1].

#### Covariates to impute MVs in the CES-D scale

The following 17 variables from the E3N database were selected as candidate risk factors of DS according to a literature review [10-12]. Sociodemographic characteristics were age, marital status, employment status, level of education, pregnancy history, menopausal status, inability to complete the eighth questionnaire alone. Psychopathological characteristics included history of depression or psychological disorder requiring treatment (collected for all questionnaires), current depression or psychological disorder requiring treatment, psychotropic drug use (collected at the fourth, sixth and seventh questionnaires), depression, anxiety or tears at menopause. Lifestyle characteristics known to be associated with DS were included: alcohol intake (in grams per day, g/d) extracted from a dietary questionnaire [15], smoking (current, former, or nonsmoker at time of the questionnaire) and sleep duration. Recent hospitalizations in a general or psychiatric hospital as well as self-report of chronic diseases were also included as risk factors for DS. The Delphi method was used to select, from the 80 chronic diseases collected, those most likely to impact on DS [16,17]. A panel of 40 physicians was constituted and was asked to classify each disease as having (i) no impact on DS, (ii) an impact on DS if it occurred recently (during the previous two years only), or (iii) an impact for lifetime occurrence. Two rounds were performed. Selected diseases were combined into a single ordinal variable representing the number of chronic diseases.

#### Qualitative study

A qualitative study was conducted to examine the hypotheses concerning the mechanism of MVs. In February 2010, a random sample of 204 women from the 71412 responders to the 8^th^ questionnaire in 2005 and for whom a telephone number was available, were invited to fill in the CES-D scale via a postal letter. The same template for the CES-D scale as for the 8^th^ questionnaire was used. Women who returned the questionnaires with MVs were systematically contacted by telephone. A random sample of complete responders was also contacted to serve as a control. The interview was semi-directive and standardized. The following open questions were systematically asked to both groups (women with and without MVs) in order to identify whether women had difficulties filling in the CES-D scale and if so whether these difficulties were independent or possibly related to depressive symptoms: How did the woman perceive the CES-D questionnaire? What did she think about the format of the questionnaire? And its content? How did she evaluate her ability and ease to express her emotions or feelings in different conditions (with her relatives, her friends, and her general practitioner)? All interviews were performed by NR during the 3 weeks following training with a psycho-oncologist at Institut Curie. Answers and comments on the sequence of questions were transcribed during the interviews. An analysis grid was then developed from the records to identify the main difficulties encountered when filling in the CES-D scale. When women wrote comments directly on the CES-D questionnaire, this information was used as a source. When several difficulties were reported, the first difficulty to be reported was used for analysis.

### *Statistical methods*

#### Internal and external validity of the data

##### Study population

Women’s characteristics were described according to the number of MVs in the CES-D scale.

##### DS among complete cases

To validate the CES-D measurement on the E3N cohort, 17 risk factors of DS were preselected from literature [10-12] and a bivariate analysis was conducted to assess the association between DS and the preselected risk factors. A negative binomial regression model was used to estimate relative risks when analyzing the score on the CES-D scale as overdispersed count data. A logistic regression model was used to estimate odds ratios when analyzing hDS as a binary variable. This binary variable was analysed on two different populations: (i) complete cases, (ii) women for whom the presenting / not presenting hDS status could be determined even in the presence of MVs.

##### Psychometric properties of the CES-D scale

We also explored the structure of the CES-D scale that has been reported to be essentially one-dimensional and the reliability computing principal component analysis, factor analysis and Cronbach’s alpha.

#### Investigation of the mechanism of MVs: a qualitative assessment

The proportion of incomplete observations, the prevalence of hDS according to the 8^th^ questionnaire and to the qualitative study were compared using chi-square tests. Potential reasons for incomplete responders were identified and described.

#### Estimation of prevalence of hDS taking MVs into account

##### Multiple imputation and imputation models

We mainly focused on multiple imputation to take into account MVs in the CES-D scale under either the hypothesis of MAR or MNAR data. MVs were imputed by using the MICE (Multivariate Imputation by Chained Equations) algorithm and R package [18], which allows for building up an imputation model with mixed-type covariates. Five imputations were performed. First, imputation model was constructed for the overall score using linear regression (pmm method) and another for multiple imputation of the hDS status using a logistic regression model (logreg method) with all preselected risk factors of DS. Second, four imputation models were constructed for multiple imputations of the items of the CES-D scale. Two different mean structures were investigated; the parsimonious model only included the CES-D items. The full model included the CES-D items and all preselected 17 candidate risk factors of DS. For each mean structure, a linear regression model (pmm method) as well as a polytomous unordered regression model (polyreg method) were used to impute missing values for the items.

##### Simulation study

A simulation study was performed to evaluate the predictive accuracy of the imputation model. Random MVs were created under a MCAR mechanism from the 39,393 complete cases. The proportion of subjects with simulated MVs corresponded to the observed prevalence of MVs on the overall population. All incomplete observations created had the same number of MVs and situations ranging from one to 19 MVs were investigated. MVs were then imputed using either single imputation (person-mean approach, each MV is replaced by the mean score for the subject) or multiple imputation (pmm and polyreg methods). Descriptive indicators were measured on the various data sets defined by the number of MVs and the imputation method, *i.e.* (i) mean and variance of the CES-D score, (ii) standard error of the mean CES-D score, (iii) prevalence of hDS.

##### CES-D score and prevalence of hDS under the ignorable MVs hypothesis

Complete case analysis, single and multiple imputation [8] approaches were performed. Three single imputation approaches were used and were denoted: minimum (each MV is imputed to 0), maximum (each MV is imputed to 3) and person-mean. We compared the results of these different approaches by using estimates of the mean and standard deviation of the CES-D score, standard error of the mean CES-D score and the prevalence of hDS. These estimates were computed in subjects with zero to four MVs, with zero to ten MVs and with zero to twenty MVs.

##### Sensitivity analysis under the nonignorable MVs hypothesis

Last, a sensitivity analysis was performed to explore the possible bias due to MNAR data. Imputation was performed according to different scenarios, using a mixture modeling approach which assumed that the variables of interest have different distributions according to the missing / non missing status. The principle of this sensitivity analysis has been previously described [19]. Briefly, we proposed a 3-step strategy:

– Fit an imputation model assuming ignorable MVs;

– Modify the imputation model by adding a parameter (expressed as the odds ratio comparing the odds of a response category among subjects with MV with those without MV for categorical variables; as the difference in expected values for continuous variables);

– Impute MVs under the scenario thus specified.

The scenarios were based on the assumption that nonresponders were more likely to present DS. Different sizes of variation were explored. Some scenarios proposed larger size of variation for positive items than for negative items as they are less difficult to answer than the negative ones, especially when considering the highest of the four response categories [20].

All analyses were performed using R software [21], multiple imputation was performed using the mice R package [22], and multiple imputation under MNAR hypothesis was performed using the SensMice R package [19].

## Results

### Internal and external validity of the data

#### Study population

**Figure 1:**
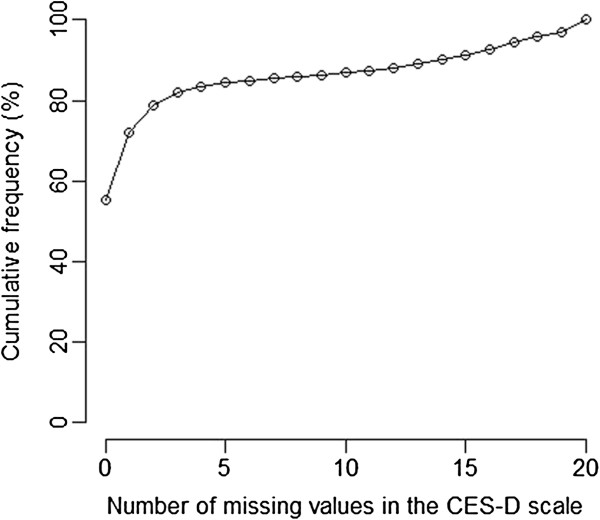
Distribution of the number of missing values in the CES-D scale (N = 71,412).

The eighth questionnaire was sent to 94,503 women: 71,412 women returned the questionnaire (response rate: 75.6%). Descriptive analyses are summarized in tables contained in Additional files 1, 2, 3. Women with 11 to 20 MVs on the CES-D scale more closely resembled complete cases in terms of psychological characteristics and morbidities than women with five to 10 MVs. The MVs distribution in the CES-D scale is summarized in Figure 1.

#### DS among complete cases

Among complete cases (N = 39,393, *i.e.* 55% of the study population), the mean CES-D score was 11.89 (standard deviation: 8.20), and the prevalence of hDS was 26.1%. Tables contained in Additional files 4, 5, 6 summarize the association with the various characteristics studied among complete cases. We found consistent results with literature. Psychological characteristics were the most strongly associated with hDS.

In the group of women in whom the presenting / not presenting hDS status could be determined, the same associations as measured by odds ratios were observed, but of greater magnitude (data not shown). Similar results were obtained for the score with a negative binomial model estimating relative risks (data not shown).

#### Psychometric properties of the CES-D scale

Principal component analysis showed the existence of a major first eigenvalue, corroborating the rather unidimensional structure of the scale. The reliability was high (Cronbach alpha coefficient = 0.89).

### Investigation of the mechanism of MVs: a qualitative assessment

A total of 183 CES-D questionnaires for the qualitative study (Q_QS_) were returned, *i.e.* a response rate of 90%: 34 (18.6%) presented at least one MV on the CES-D scale. MVs were observed for each item. The most frequently missing item was “I felt that I was just as good as other people” (13 MVs), and the least frequently missing one was “I felt depressed” (one MV).

The prevalence of hDS was 30.2% among complete responders. Imputation of all the MVs successively to 0 and 3 gave prevalence of hDS between 41.2% and 64.7% among women who had MVs in the scale, and between 32.2% and 36.6% among the whole sample (Table 1). Although the differences in prevalence were not statistically significant, these intervals did not include the point estimate based on complete data only, suggesting that MVs were not MCAR. Being an incomplete responder at Q8 did not appear to be associated with being an incomplete responder at Q_QS_ (p = 0.547). However, the presenting / not presenting hDS status at Q8 was associated with this status at Q_QS_ (p < 0.001).

**Table 1:** Numbers and prevalence of incomplete observations and high depressive symptoms among women included in the qualitative study (N = 183)

		**CES-D scale (Qualitative study)**		
		**No MV**	**At least one MV**	**All**
CES-D scale	NhDS	104	12	116
(Qualitative study)	hDS	45	14	59
	Undetermined	-	8	8
CES-D scale	No MV	91 (82.7%)	19 (17.3%)	110 (100.0%)
(8^th ^questionnaire)	At least one MV	57 (79.2%)	15 (20.8%)	72 (100.0%)
		NhDS	hDS	All
CES-D scale	NhDS	79 (77.5%)	23 (22.5%)	102 (100.0%)
(8^th ^questionnaire)	hDS	19 (43.2%)	25 (56.8%)	44 (100.0%)

Abbreviations: hDS, Presenting high depressive symptoms (CES-D score ≥ 16); MV, Missing Value; NhDS, Not presenting high depressive symptoms.

Sixteen women of the 183 added written comments on the questionnaires, and among them, the eleven complete responders were not called but their written comments were analysed together with other controls. None of the women contacted refused to be interviewed. None of them declared that they were reluctant to answer the CES-D questions. Four main types of potential reasons for nonresponses were identified (Table 2). These results suggested that the missingness mechanism was not compatible with the hypothesis of MCAR data. The hypotheses of MAR and MNAR data both remained plausible.

**Table 2:** Description of the participants’ responses to the qualitative study

	**No MV**	**At least one MV**	**All**
Postal letter			
Potential reason for MV (given in writing)			
Personal physical disorders	5	3	8
Personal psychological disorders	2	1	3
Stressful life event	1	0	1
Relative's disease or death	3	1	4
No potential reason for MV	138	29	167
*Total (participants)*	*149*	*34*	*183*
Interview study			
Potential reason for MV (given orally)			
Personal physical disorders	1	3	4
Personal psychological disorders	2	3	5
Stressful life event	0	4	4
Relative's disease or death	3	4	7
No potential reason for MV	21	15	36
*Total (contacted)*	*27*	*29*	*56*

Abbreviation: MV, Missing Value.

### Estimation of prevalence taking missing data into account

#### Simulation study

MVs were randomly created for 44.8% of the complete cases, corresponding to the rate of missing data observed in the cohort. Imputation methods pmm and polyreg, based on a linear regression model and a polytomous unordered regression model, respectively, gave similar results, up to a large number of MVs (see Additional file 7). The cut-off of 4 MVs did not appear to be associated with any particularly interesting properties. Below 15 MVs, multiple imputation performed very well, while single imputation gave some biased results.

#### CES-D score and prevalence of hDS under the ignorable MVs hypothesis

The CES-D score and the prevalence of hDS were described (Table 3). Both complete cases and person-mean imputation gave biased results compared to multiple imputation of the items. Biases were observed for both the total score and the presenting / not presenting hDS status. The two regression models studied for imputation of the values of the items (linear regression model and polytomous unordered regression model) gave similar results. Addition of covariates to the imputation model for items did not substantially modify the results.

**Table 3:** Score on the CES-D scale and prevalence of high depressive symptoms according to various methods of handling missing values assuming ignorable missing data

				**Score on the CES-D scale**		
		**N**	**Mean**	**SD**	**SEM**	**≥16 (%)**
Complete cases		39,393	11.89	8.20	0.04	26.09
Classifiable cases		55,964	-	-		30.36
Single imputation - Minimum value						
	0 - 20 MV	71,412	11.02	8.28	0.03	23.79
	0 - 10 MV	62,053	12.10	8.20	0.03	27.06
	0 - 4 MV	59,562	12.07	8.22	0.03	26.91
Single imputation - Maximum value						
	0 - 20 MV	71,412	19.68	16.15	0.06	45.42
	0 - 10 MV	62,053	14.53	9.62	0.04	37.19
	0 - 4 MV	59,562	13.71	8.74	0.04	34.58
Single imputation - Person mean						
	0 - 19 MV	69,242	13.81	10.16	0.04	33.16
	0 - 10 MV	62,053	12.76	8.82	0.04	28.99
	0 - 4 MV	59,562	12.45	8.52	0.03	27.78
Multiple imputation						
Score						
	0 - 20 MV	71,412	12.23	8.37	0.04	27.58
	0 - 10 MV	62,053	12.12	8.33	0.04	27.10
	0 - 4 MV	59,562	12.06	8.29	0.05	26.81
Status ≥ 16						
	0 - 20 MV	71,412	-	-		31.04
	0 - 10 MV	62,053	-	-		30.33
	0 - 4 MV	59,562	-	-		29.27
Multiple imputation						
Items						
Parsimonious model						
pmm method						
	0 - 20 MV	71,412	13.30	9.02	0.04	31.99
	0 - 10 MV	62,053	12.76	8.71	0.04	29.75
	0 - 4 MV	59,562	12.48	8.47	0.03	28.62
polyreg method						
	0 - 20 MV	71,412	13.18	9.05	0.04	31.54
	0 - 10 MV	62,053	12.76	8.72	0.04	29.75
	0 - 4 MV	59,562	12.47	8.48	0.03	28.61
Full model						
pmm method						
	0 - 20 MV	71,412	13.26	9.04	0.03	31.85
	0 - 10 MV	62,053	12.76	8.72	0.04	29.74
	0 - 4 MV	59,562	12.48	8.48	0.03	28.63
polyreg method						
	0 - 20 MV	71,412	13.22	9.05	0.04	31.73
	0 - 10 MV	62,053	12.76	8.72	0.04	29.80
	0 - 4 MV	59,562	12.48	8.48	0.03	28.63

Abbreviation: MV, Missing value; SEM, Standard error of the mean.

#### Sensitivity analysis under the nonignorable MVs hypothesis

Results concerning the prevalence of hDS after imputation of the values of the items considered as qualitative and as quantitative variables and the presenting / not presenting hDS status are presented in Table 4 and tables contained in Additional files 8 and 9, respectively.

**Table 4:** Score on the CES-D Scale and prevalence of high depressive symptoms after imputation of the values for items considered as qualitative variables, according to various scenarios of nonignorable missing data

		**Parsimonious model**			**Full model**		
		**Score on the CES-D scale**			**Score on the CES-D scale**		
	**N**	**Mean**	**SD**	**≥16 (%)**	**Mean**	**SD**	**≥16 (%)**
Scenario 1							
θ^a^ = (1.2; 1.5; 2.0) for all items							
0 - 20 MV	71,412	13.43	9.18	32.49	13.47	9.19	32.71
0 - 10 MV	62,053	12.84	8.77	30.05	12.84	8.77	30.07
0 - 4 MV	59,562	12.52	8.50	28.81	12.53	8.50	28.83
Scenario 2:							
θ^a ^= (1.2; 1.5; 2.0) for N items, θ^a ^= (1.5; 2.0; 2.5) for P items							
0 - 20 MV	71,412	13.45	9.18	32.57	13.50	9.19	32.79
0 - 10 MV	62,053	12.85	8.77	30.07	12.85	8.77	30.10
0 - 4 MV	59,562	12.53	8.50	28.82	12.53	8.50	28.84
Scenario 3							
θ^a ^= (2.0; 3.0; 5.0) for N items, θ^a ^= (3.0; 5.0; 8.0) for P items							
0 - 20 MV	71,412	13.88	9.37	34.41	13.92	9.39	34.66
0 - 10 MV	62,053	12.97	8.82	30.58	12.98	8.82	30.59
0 - 4 MV	59,562	12.62	8.52	29.12	12.62	8.52	29.19
Scenario 4							
θ^a ^= (4.0; 6.0; 10.0) for N items, θ^a ^= (6.0; 10.0; 15.0) for P items							
0 - 20 MV	71,412	14.19	9.47	36.17	14.24	9.50	36.39
0 - 10 MV	62,053	13.06	8.84	30.98	13.06	8.84	30.98
0 - 4 MV	59,562	12.68	8.52	29.42	12.68	8.52	29.44

Abbreviations: MV, Missing value; N items, negative items; P items, positive items.

a: Vector of parameters for the MNAR scenario, each parameter corresponding to the odds ratio expressing the excess risk to respond one of the response categories (*i.e. *an item score of one, two or three) compared to the reference (i.e. an item score of zero), in subjects with MV compared to subjects without MV.

As expected, the impact on prevalence was proportional to the modeled shifts. For the most extreme scenario considered here and when items were treated as qualitative variables (Table 4), the prevalence of hDS increased from 31.7% (multiple imputation assuming MAR data) to 36.4% (4.6% increase) when including the whole study population. Addition of the covariates to the imputation model for items did not substantially modify the results. This was expected, as no hypothesis had been proposed on the imputation model for the association between the missingness mechanism and the covariates. Similar results were observed when considering items as quantitative variables.

## Discussion

Many population-based studies have been conducted to estimate the prevalence of psychiatric disorders based on self-rated scales or to investigate associations with these disorders. Few of these studies have assessed the impact of missing data on the accuracy of estimates. The present study investigated MVs in the CES-D scale, a validated and easy-to-use tool, commonly used to identify DS. A qualitative study showed that none of the women contacted declared that they were reluctant to answer the questions of the CES-D scale, suggesting that the missingness mechanism could be ignorable. Multiple imputation is then an adequate approach to handle missing data. An imputation model including the CES-D items and various covariates was shown to have the same predictive properties as a model using the CES-D items only. A simulation study showed that multiple imputation performed well, even in the presence of a large amount of MVs and using an imputation model including only CES-D items. The prevalence of hDS was 26.1% among complete cases, 30.4% among classifiable cases, and 31.7% among all women after multiple imputation. In a sensitivity analysis, these estimates were found to be quite robust under plausible MNAR scenarios.

The rate of incomplete responses for the CES-D scale in the E3N cohort was about 45%, a much higher rate than in previous publications, in which fewer than 10% of responders to the scales presented MVs [5,23-28]. Various aspects related to both the survey design and the presentation of the questionnaire [23-26,29,30] could explain this substantial incomplete response rate. Elderly people are more likely to be incomplete responders [31], which was confirmed on our cohort, in which MVs were more often observed in older women. The length of the questionnaire in which the CES-D scale was included probably also accounted for this high rate. In fact, the proportion of missing data in the Q_QS_ (one page) was 19% compared to 45% in the 11 pages of the Q8. High levels of MVs have been attributed to an increase in socially desirable responses to the content of the questions, especially in elderly population [32,33]. However, in the present qualitative study, despite the age of the women interviewed, none of them declared that they had been embarrassed by the questions. The qualitative study casts some light on the recurrent issue of the missingness mechanism. Interviewing “cases” as well as “controls” allowed us to compare the responses and consequently improve interpretability of our survey. This type of qualitative study should be carried out more often to investigate the missingness mechanism that cannot be tested from the data.

To address MVs in depressive scales, it is common practice to apply single imputation to the person-mean value and to exclude the whole observation when the proportion of MVs in a subject exceeds a given cut-off (typically 10 to 20%) [5,27,31]. Excluding observations has been repeatedly shown to lead to biased results when MVs are not MCAR. Completers of the CES-D scale have been found to be different from non-completers [25-27,31,32] (*e.g.* in terms of age, socioeconomic status, reported health status) and the MCAR assumption therefore appears to be irrelevant.

Due to the absence of evidence for a MNAR mechanism, multiple imputation is an attractive alternative. This generic technique can be applied to virtually any missing data situation [18,34-36] and is now available in standard statistical software [6,37,38]. This technique was also the most accurate method for dealing with missing data for the Zung Self-Reported depression scale [39]. Selection of the best imputation model intimately depends on variables collected. However, the results obtained after multiple imputation based on the parsimonious model were surprisingly similar to those obtained with the full model, even in presence of scarce data for the CES-D scale. This can be explained by the unidimensionality of the CES-D scale that we also measured. All items of the scale measure the same concept and are the best predictors of items presenting MVs. Other variables add very little additional information for the estimation of the CES-D score. This also entails that the same imputation model is probably suitable whatever the number of missing items in the CES-D scale. Last, in regression analyses of the association between an outcome and the CES-D scale, it is recommended that the imputation model be adjusted for the CES-D items and for all other variables in the analysis model in order to limit the risk of bias in the association measure [18,40].

Predictive mean matching based on linear regression and polytomous unordered regression from the mice package gave very similar results when imputing MVs under the MAR assumption. From a computational point of view, the former has the advantage of being the fastest, and easiest to fit on small sample sizes. Nevertheless, this model requires a very strong assumption of linearity that must be carefully checked.

An MNAR mechanism can never be ruled out. Nonignorability implies a difference between responders and nonresponders even after taking all observed covariates into account. Both possibilities were then considered in the present study in a sensitivity analysis. Although often recommended, sensitivity analyses are too rarely performed. They should be considered more often to assess the robustness of the results by studying how variations in the imputation model impact on the overall results. We formulated various scenarios in which the MVs for items were MNAR. It was reassuring that the estimated prevalence of hDS was only slightly modified: in the most extreme case investigated, the absolute difference in prevalence was less than 5% on the whole population.

After multiple imputation under the MAR hypothesis, the prevalence of hDS in the present study was 31.7% (14.3% when considering the cut-off of 23 which was recommended by Fuhrer & Rouillon [41] to define high depressive symptoms among French women). This estimate is relatively high, but the results are difficult to compare with previous findings due to lack of information about MVs and the diversity of endpoints when assessing DS. Two population-based studies conducted in France estimated a prevalence of major depression during the past 12 months of 7.8% and 5.0%, and a prevalence of severe major depression of 3.2% and 2.6%, with a refusal rate of 42.1% and 37.0%, respectively [30]. A cross-sectional survey conducted in several European countries estimated the 12-month and lifetime prevalence rates of major depression in France at 6.0% and 21.4%, respectively, with a participation rate of 46% [42].

A review based on elderly Caucasians reported a prevalence of hDS from 7.2% to 49% [11], but major discrepancies were observed according to cross-cultural and geographic variations. An older review in an elderly population reported an average prevalence of hDS of 13.5% (range: 4 to 35%), with higher prevalence rates for women [10].

Participants included in this analysis did not constitute a representative sample of the general population, as all subjects were women older than 55 with high socioeconomic status, presenting a large number of chronic diseases and a high prevalence of recent hospitalization, and reporting a high prevalence of history or current psychological disorders and psychotropic drug use. Although these differences may result in overestimation of the prevalence of hDS in the general population, they are unlikely to alter the findings on how to handle MVs in the CES-D scale.

## Conclusions

In conclusion, we recommend considering MVs in items of the CES-D scale as ignorable and the use of multiple imputation to perform all analyses. The imputation model can be restricted to the available CES-D items responses for score estimate and to the CES-D items and the covariates in regression analyses. However, based on the R package developed [19], it is worth investigating the robustness of any descriptive or etiologic analysis under plausible scenarios for MNAR data. Methods used in the present study on the CES-D scale can be applicable to other rating scales when dealing with MVs. If MVs can be considered as ignorable, then multiple imputation is recommended.

## Abbreviations

CES-D scale: Center for Epidemiologic Studies Depression scale; DS: Depressive symptoms; E3N: Etude Epidémiologique auprès des femmes de la Mutuelle Générale de l’Education Nationale; hDS: High depressive symptoms (score on the CES-D scale >16); IS: Interview study; MAR: Missing at random; MCAR: Missing completely at random; MNAR: Missing not at random; MVs: Missing values.

## Competing interests

The authors declared that they have no competing interest.

## Authors’ contributions

NR performed all statistical analyses and interviews, and wrote the first draft of the manuscript. HV and RG contributed to methods and interpretation of data (areas of expertise: psychiatry and statistics, respectively). FCC conceived the E3N cohort and supervised the data collection. XP designed the study, contributed to methods, analysis and interpretation of the data, and participated in drafting the manuscript. All authors read, commented and approved the final version of the manuscript.

## Pre-publication history

The pre-publication history for this paper can be accessed here:

http://www.biomedcentral.com/1471-2288/13/28/prepub

## Supplementary Material

Additional file 1 Description (%) of socio-demographic variables of all women included according to the number of missing values in the CES-D scale (N = 71,412). Click here for file

Additional file 2 Description (%) of psychopathological characteristics of all women included according to the number of missing values in the CES-D scale (N = 71,412). Click here for file

Additional file 3 Description (%) of morbidities and behavioral characteristics of all women included according to the number of missing values in the CES-D scale (N = 71,412). Click here for file

Additional file 4 Prevalence of high depressive symptoms according to the variables related to socio-demographic characteristics among complete cases (N = 39,393). Click here for file

Additional file 5 Prevalence of high depressive symptoms according to the variables related to psychological characteristics among complete cases (N = 39,393). Click here for file

Additional file 6 Prevalence of high depressive symptoms according to the variables related to morbidities and behavioral characteristics among complete cases (N = 39,393). Click here for file

Additional file 7 **Results of the simulation study evaluating the predictive accuracy of variouds methods in the case of ignorable MVs. **In blue: pmm method – multiple imputation based on a linear regression imputation model. In red: polyreg method – multiple imputation based on a polytomous unordered regression model. In green: single imputation based on the person-mean approach. The dotted lines correspond to the “true” values observed before simulating MVs. Click here for file

Additional file 8 Score on the CES-D scale and prevalence of high depressive symptoms after imputation of the values for items considered as quantitative variables, according to various scenarios of nonignorable missing data. Click here for file

Additional file 9 Prevalence of high depressive symptoms after imputation of the presenting / not presenting depressive symptoms status, according to various scenarios of nonignorable missing data. Click here for file
