# Supplementary material for: Dealing with missing data in the Center for Epidemiologic Studies Depression self-report scale: a study based on the French E3N cohort
Source: BMC Med Res Methodol. 2013 Feb 21;13:28. doi: 10.1186/1471-2288-13-28 (PMC3602286; doi:10.1186/1471-2288-13-28)
Supplement: Additional file 1 — Description (%) of socio-demographic variables of all women included according to the number of missing values in the CES-D scale (N = 71,412). [file 1471-2288-13-28-S1.doc]

Description (%) of socio-demographic variables of all women included according to the number of missing values in the CES-D scale (N=71,412).

|  |  | All |  | 0 MV |  | 1 to 4 MV |  | 5 to 10 MV |  | 11 to 20 MV |
| --- | --- | --- | --- | --- | --- | --- | --- | --- | --- | --- |
|  |  | (N=71,412) |  | (N=39,393) |  | (N=20,169) |  | (N=2,491) |  | (N=9,359) |
|  |  |  |  |  |  |  |  |  |  |  |
| Age | |  |  |  |  |  |  |  |  |  |
|  | < 60 y.o. | 31.4 |  | 37.3 |  | 29.7 |  | 19.2 |  | 13.3 |
|  | 60 - 65 y.o. | 25.8 |  | 27.9 |  | 25.5 |  | 21.6 |  | 18.8 |
|  | 65 - 70 y.o. | 20.9 |  | 19.5 |  | 21.9 |  | 24.1 |  | 23.9 |
|  | 70 - 75 y.o. | 13.4 |  | 10.0 |  | 14.4 |  | 18.8 |  | 23.8 |
|  | > 75 y.o. | 8.5 |  | 5.2 |  | 8.5 |  | 16.3 |  | 20.2 |
|  |  |  |  |  |  |  |  |  |  |  |
| Marital status | |  |  |  |  |  |  |  |  |  |
|  | Married / in a relationship | 70.9 |  | 72.8 |  | 70.0 |  | 63.5 |  | 66.4 |
|  | Single | 6.0 |  | 5.9 |  | 6.2 |  | 7.3 |  | 5.4 |
|  | Widowed | 10.9 |  | 8.9 |  | 11.2 |  | 15.1 |  | 17.8 |
|  | Divorced / separated | 12.0 |  | 12.2 |  | 12.4 |  | 13.8 |  | 9.9 |
|  | MV | 0.2 |  | 0.1 |  | 0.3 |  | 0.3 |  | 0.6 |
|  |  |  |  |  |  |  |  |  |  |  |
| Employment status | |  |  |  |  |  |  |  |  |  |
|  | Employed | 19.4 |  | 22.5 |  | 19.1 |  | 13.0 |  | 8.7 |
|  | Unemployed | 77.7 |  | 75.0 |  | 77.5 |  | 83.0 |  | 88.1 |
|  | MV | 2.9 |  | 2.5 |  | 3.4 |  | 4.1 |  | 3.2 |
|  |  |  |  |  |  |  |  |  |  |  |
| Level of education | |  |  |  |  |  |  |  |  |  |
|  | Less than A level | 11.7 |  | 9.4 |  | 10.3 |  | 15.5 |  | 23.2 |
|  | A level to bachelor degree | 66.9 |  | 69.5 |  | 66.4 |  | 62.4 |  | 58.1 |
|  | Master degree or higher | 17.3 |  | 17.4 |  | 18.9 |  | 17.2 |  | 13.8 |
|  | MV | 4.1 |  | 3.7 |  | 4.4 |  | 4.9 |  | 4.8 |
|  |  |  |  |  |  |  |  |  |  |  |
| Pregnancy history | |  |  |  |  |  |  |  |  |  |
|  | No children, nulligravida | 9.1 |  | 9.0 |  | 9.1 |  | 10.5 |  | 9.4 |
|  | No children, non-nulligravida | 2.4 |  | 2.5 |  | 2.4 |  | 2.7 |  | 2.2 |
|  | 1 child | 15.5 |  | 15.5 |  | 15.1 |  | 14.7 |  | 16.7 |
|  | 2 children | 43.0 |  | 44.4 |  | 42.7 |  | 39.9 |  | 38.8 |
|  | 3 children | 21.4 |  | 21.2 |  | 21.7 |  | 21.3 |  | 22.0 |
|  | 4 children and more | 7.7 |  | 6.8 |  | 8.2 |  | 9.9 |  | 9.8 |
|  | MV | 0.7 |  | 0.6 |  | 0.8 |  | 1.1 |  | 1.2 |
|  |  |  |  |  |  |  |  |  |  |  |
| Survey respondent | |  |  |  |  |  |  |  |  |  |
|  | Herself | 97.7 |  | 97.7 |  | 97.9 |  | 97.6 |  | 97.0 |
|  | Helped by another | 1.8 |  | 1.9 |  | 1.6 |  | 1.7 |  | 2.2 |
|  | Another | 0.5 |  | 0.4 |  | 0.5 |  | 0.7 |  | 0.8 |
| Menopausal status | |  |  |  |  |  |  |  |  |  |
|  | Premenopausal | 0.8 |  | 0.9 |  | 0.7 |  | 0.4 |  | 0.4 |
|  | Menopausal | 99.2 |  | 99.1 |  | 99.3 |  | 99.5 |  | 99.6 |
|  |  |  |  |  |  |  |  |  |  |  |

Abbreviation: MV, Missing Value.
