# Supplementary material for: Dealing with missing data in the Center for Epidemiologic Studies Depression self-report scale: a study based on the French E3N cohort
Source: BMC Med Res Methodol. 2013 Feb 21;13:28. doi: 10.1186/1471-2288-13-28 (PMC3602286; doi:10.1186/1471-2288-13-28)
Supplement: Additional file 2 — Description (%) of psychopathological characteristics of all women included according to the number of missing values in the CES-D scale (N = 71,412). [file 1471-2288-13-28-S2.doc]

**Description (%) of psychopathological characteristics of all women included according to the number of missing values in the CES-D scale (N=71,412).**

|  |  | All |  | 0 MV |  | 1 to 4 MV |  | 5 to 10 MV |  | 11 to 20 MV |
| --- | --- | --- | --- | --- | --- | --- | --- | --- | --- | --- |
|  |  | (N=71,412) |  | (N=39,393) |  | (N=20,169) |  | (N=2,491) |  | (N=9,359) |
|  |  |  |  |  |  |  |  |  |  |  |
| Depression, psychological disorders | |  |  |  |  |  |  |  |  |  |
| requiring treatment (Q1 - Q7) | |  |  |  |  |  |  |  |  |  |
|  | No | 68.9 |  | 70.1 |  | 67.3 |  | 60.4 |  | 69.4 |
|  | Yes | 31.1 |  | 29.9 |  | 32.7 |  | 39.6 |  | 30.6 |
|  |  |  |  |  |  |  |  |  |  |  |
| Depression, psychological disorders | |  |  |  |  |  |  |  |  |  |
| requiring treatment (Q8) | |  |  |  |  |  |  |  |  |  |
|  | No | 91.2 |  | 92.2 |  | 90.1 |  | 84.4 |  | 91.2 |
|  | Yes | 8.8 |  | 7.8 |  | 9.9 |  | 15.6 |  | 8.8 |
|  |  |  |  |  |  |  |  |  |  |  |
| Sleeping pill use | |  |  |  |  |  |  |  |  |  |
| (> 3 times/week) | |  |  |  |  |  |  |  |  |  |
|  | No | 88.6 |  | 90.5 |  | 87.8 |  | 81.7 |  | 84.0 |
|  | Yes | 10.5 |  | 8.7 |  | 11.4 |  | 17.0 |  | 14.5 |
|  | MV | 0.9 |  | 0.8 |  | 0.8 |  | 1.3 |  | 1.5 |
|  |  |  |  |  |  |  |  |  |  |  |
| Psychotropic drug use | |  |  |  |  |  |  |  |  |  |
| (> 3 times/week) | |  |  |  |  |  |  |  |  |  |
|  | No | 82.5 |  | 83.9 |  | 81.4 |  | 73.9 |  | 80.9 |
|  | Yes | 16.6 |  | 15.3 |  | 17.8 |  | 24.8 |  | 17.7 |
|  | MV | 0.9 |  | 0.8 |  | 0.8 |  | 1.3 |  | 1.5 |
|  |  |  |  |  |  |  |  |  |  |  |
| Depression, anxiety, | |  |  |  |  |  |  |  |  |  |
| tears at menopause | |  |  |  |  |  |  |  |  |  |
|  | No | 76.9 |  | 78.2 |  | 76.9 |  | 72.8 |  | 72.3 |
|  | Yes | 19.6 |  | 20.1 |  | 20.5 |  | 22.8 |  | 15.0 |
|  | MV | 3.5 |  | 1.7 |  | 2.6 |  | 4.3 |  | 12.7 |
|  |  |  |  |  |  |  |  |  |  |  |

Abbreviation: MV, Missing Value.
