# Supplementary material for: Dealing with missing data in the Center for Epidemiologic Studies Depression self-report scale: a study based on the French E3N cohort
Source: BMC Med Res Methodol. 2013 Feb 21;13:28. doi: 10.1186/1471-2288-13-28 (PMC3602286; doi:10.1186/1471-2288-13-28)
Supplement: Additional file 3 — Description (%) of morbidities and behavioral characteristics of all women included according to the number of missing values in the CES-D scale (N = 71,412). [file 1471-2288-13-28-S3.doc]

Description (%) of morbidities and behavioral characteristics of all women included according to the number of missing values in the CES-D scale (N=71,412).

|  |  |  | All |  | 0 MV |  | 1 to 4 MV |  | 5 to 10 MV |  | 11 to 20 MV |
| --- | --- | --- | --- | --- | --- | --- | --- | --- | --- | --- | --- |
|  |  |  | (N=71,412) |  | (N=39,393) |  | (N=20,169) |  | (N=2,491) |  | (N=9,359) |
|  |  |  |  |  |  |  |  |  |  |  |  |
| Chronic diseases | |  |  |  |  |  |  |  |  |  |  |
|  | 0 |  | 28.0 |  | 29.6 |  | 27.3 |  | 21.4 |  | 25.1 |
|  | 1 |  | 35.2 |  | 36.1 |  | 34.7 |  | 30.5 |  | 33.7 |
|  | 2 |  | 21.9 |  | 21.4 |  | 22.2 |  | 24.3 |  | 22.5 |
|  | 3 |  | 9.4 |  | 8.5 |  | 9.8 |  | 13.6 |  | 10.9 |
|  | 4 or more |  | 5.5 |  | 4.4 |  | 6.0 |  | 10.2 |  | 7.7 |
|  |  |  |  |  |  |  |  |  |  |  |  |
| Recent hospitalization (<2y) | |  |  |  |  |  |  |  |  |  |  |
|  | No |  | 81.4 |  | 82.6 |  | 80.7 |  | 77.4 |  | 79.2 |
|  | Yes |  | 18.6 |  | 17.4 |  | 19.3 |  | 22.6 |  | 20.8 |
|  |  |  |  |  |  |  |  |  |  |  |  |
| Alcohol intake (g/d) | |  |  |  |  |  |  |  |  |  |  |
|  | 0 |  | 11.9 |  | 10.4 |  | 11.4 |  | 15.9 |  | 18.2 |
|  | 0 - 2 |  | 15.4 |  | 15.5 |  | 15.2 |  | 15.6 |  | 15.4 |
|  | 2 - 4 |  | 10.6 |  | 11.0 |  | 10.7 |  | 10.2 |  | 8.7 |
|  | 4 - 8 |  | 15.2 |  | 16.0 |  | 15.1 |  | 14.1 |  | 12.2 |
|  | 8 - 16 |  | 20.9 |  | 21.7 |  | 20.9 |  | 18.6 |  | 18.0 |
|  | 16 - 32 |  | 17.3 |  | 17.7 |  | 17.6 |  | 16.1 |  | 15.4 |
|  | > 32 |  | 7.0 |  | 6.7 |  | 7.5 |  | 7.2 |  | 6.7 |
|  | MV |  | 1.8 |  | 1.0 |  | 1.6 |  | 2.4 |  | 5.4 |
|  |  |  |  |  |  |  |  |  |  |  |  |
| Smoking | |  |  |  |  |  |  |  |  |  |  |
|  | Non-smoker |  | 52.3 |  | 52.2 |  | 50.6 |  | 52.8 |  | 56.1 |
|  | Former smoker | | 39.3 |  | 40.1 |  | 41.1 |  | 37.4 |  | 32.5 |
|  | Current smoker | | 6.8 |  | 7.1 |  | 7.1 |  | 6.5 |  | 5.3 |
|  | MV |  | 1.6 |  | 0.6 |  | 1.2 |  | 3.4 |  | 6.1 |
|  |  |  |  |  |  |  |  |  |  |  |  |
| Sleep time (/day) | |  |  |  |  |  |  |  |  |  |  |
|  | < 6 hours |  | 2.8 |  | 2.4 |  | 2.8 |  | 4.7 |  | 4.3 |
|  | 6 - 7 hours |  | 12.7 |  | 11.9 |  | 13.4 |  | 15.1 |  | 14.1 |
|  | 7 - 8 hours |  | 28.8 |  | 29.6 |  | 29.0 |  | 27.6 |  | 25.4 |
|  | 8 - 9 hours |  | 35.6 |  | 37.7 |  | 34.6 |  | 30.3 |  | 30.5 |
|  | 9 - 10 hours |  | 12.3 |  | 12.5 |  | 12.3 |  | 10.7 |  | 11.7 |
|  | 10 hours and more | | 4.2 |  | 3.7 |  | 4.5 |  | 5.7 |  | 5.1 |
|  | MV |  | 3.6 |  | 2.2 |  | 3.4 |  | 6.0 |  | 9.0 |
|  |  |  |  |  |  |  |  |  |  |  |  |

Abbreviation: MV, Missing Value.
