# Supplementary material for: Dealing with missing data in the Center for Epidemiologic Studies Depression self-report scale: a study based on the French E3N cohort
Source: BMC Med Res Methodol. 2013 Feb 21;13:28. doi: 10.1186/1471-2288-13-28 (PMC3602286; doi:10.1186/1471-2288-13-28)
Supplement: Additional file 4 — Prevalence of high depressive symptoms according to the variables related to socio-demographic characteristics among complete cases (N = 39,393). [file 1471-2288-13-28-S4.doc]

Prevalence of high depressive symptoms according to the variables related to socio-demographic characteristics among complete cases (N=39,393).

|  |  |  |  | Prevalence of high depressive symptoms | | | | |
| --- | --- | --- | --- | --- | --- | --- | --- | --- |
|  |  | N | N hDS | % hDS | 95% CI | OR | 95% CI | *P*^a^ |
|  |  |  |  |  | |  | |  |
| Age |  |  |  |  | |  | | <0.001 |
|  | < 60 y.o. | 14,712 | 3,746 | 25.5 | 24.8, 26.2 | 1 |  |  |
|  | 60 - 65 y.o. | 10,996 | 2,814 | 25.6 | 24.8, 26.4 | 1.01 | 0.95, 1.07 |  |
|  | 65 - 70 y.o. | 7,683 | 2,033 | 26.5 | 25.5, 27.5 | 1.05 | 0.99, 1.12 |  |
|  | 70 - 75 y.o. | 3,949 | 1,086 | 27.5 | 26.1, 28.9 | 1.11 | 1.03, 1.20 |  |
|  | > 75 y.o. | 2,053 | 599 | 29.2 | 27.2, 31.2 | 1.21 | 1.09, 1.34 |  |
|  |  |  |  |  | |  | |  |
| Marital status | |  |  |  | |  | | <0.001 |
|  | Married / in a relationship | 28,687 | 6,780 | 23.6 | 23.1, 24.1 | 1 |  |  |
|  | Single | 2,343 | 695 | 29.7 | 27.8, 31.6 | 1.36 | 1.24, 1.49 |  |
|  | Widowed | 3,519 | 1,232 | 35.0 | 33.4, 36.6 | 1.74 | 1.62, 1.87 |  |
|  | Divorced / separated | 4,791 | 1,550 | 32.4 | 31.0, 33.7 | 1.55 | 1.45, 1.65 |  |
|  | MV | 53 | 21 | 39.6 | 26.5, 54.0 | - |  |  |
|  |  |  |  |  | |  | |  |
| Employment status | |  |  |  | |  | | 0.471 |
|  | Employed | 8,869 | 2,286 | 25.8 | 24.9, 26.7 | 1 |  |  |
|  | Unemployed | 29,540 | 7,727 | 26.2 | 25.7, 26.7 | 1.02 | 0.97, 1.08 |  |
|  | MV | 984 | 265 | 26.9 | 24.2, 29.8 | - |  |  |
|  |  |  |  |  | |  | |  |
| Level of education | |  |  |  | |  | | <0.001 |
|  | Less than A level | 3,714 | 1,194 | 32.1 | 30.6, 33.7 | 1 |  |  |
|  | A level to bachelor degree | 27,375 | 7,136 | 26.1 | 25.5, 26.6 | 0.74 | 0.69, 0.80 |  |
|  | Master degree or higher | 6,849 | 1,536 | 22.4 | 21.4, 23.4 | 0.61 | 0.56, 0.67 |  |
|  | MV | 1,455 | 412 | 28.3 | 26.0, 30.7 | - |  |  |
|  |  |  |  |  | |  | |  |
| Pregnancy history | |  |  |  | |  | | <0.001 |
|  | No children, nulligravida | 3,559 | 982 | 27.6 | 26.1, 29.1 | 1 |  |  |
|  | No children, non-nulligravida | 9,82 | 297 | 30.2 | 27.4, 33.2 | 1.14 | 0.97, 1.33 |  |
|  | 1 child | 6,109 | 1,780 | 29.1 | 28.0, 30.3 | 1.08 | 0.98, 1.18 |  |
|  | 2 children | 17,495 | 4,483 | 25.5 | 25.0, 26.3 | 0.90 | 0.83, 0.98 |  |
|  | 3 children | 8,344 | 2,059 | 24.7 | 23.8, 25.6 | 0.86 | 0.79, 0.94 |  |
|  | 4 children and more | 2,682 | 611 | 22.8 | 21.2, 24.4 | 0.77 | 0.69, 0.87 |  |
|  | MV | 222 | 66 | 29.7 | 23.8, 36.2 | - |  |  |
|  |  |  |  |  | |  | |  |
| Survey respondent | |  |  |  | |  | | 0.476 |
|  | Herself | 38,483 | 10,041 | 26.1 | 25.7, 26.5 | 1 |  |  |
|  | Helped by another | 735 | 185 | 25.2 | 22.1, 28.5 | 0.95 | 0.81, 1.13 |  |
|  | Another | 175 | 52 | 29.7 | 23.1, 37.1 | 1.20 | 0.87, 1.66 |  |
|  |  |  |  |  | |  | |  |
| Menopausal status | |  |  |  | |  | | 0.331 |
|  | Premenopausal | 365 | 83 | 22.7 | 18.5, 27.4 | 1 |  |  |
|  | Menopausal | 39,024 | 10,194 | 26.1 | 25.7, 26.6 | 1.20 | 0.94, 1.54 |  |
|  |  |  |  |  | |  | |  |

Abbreviations: hDS, Presenting high depressive symptoms (CES-D score≥16); MV, Missing Value; N: Number of women.

^a^ Two sided p value for the overall likelihood ratio test
