# Supplementary material for: Dealing with missing data in the Center for Epidemiologic Studies Depression self-report scale: a study based on the French E3N cohort
Source: BMC Med Res Methodol. 2013 Feb 21;13:28. doi: 10.1186/1471-2288-13-28 (PMC3602286; doi:10.1186/1471-2288-13-28)
Supplement: Additional file 5 — Prevalence of high depressive symptoms according to the variables related to psychological characteristics among complete cases (N = 39,393). [file 1471-2288-13-28-S5.doc]

Prevalence of high depressive symptoms according to the variables related to psychological characteristics among complete cases (N=39,393).

|  |  |  |  | Prevalence of high depressive symptoms | | | | |
| --- | --- | --- | --- | --- | --- | --- | --- | --- |
|  |  | N | N hDS | % hDS | 95% CI | OR | 95% CI | *P*^a^ |
|  |  |  |  |  | |  | |  |
| Depression, psychological disorders | | |  |  | |  | |  |
| requiring treatment (Q1 - Q7) | |  |  |  | |  | | <0.001 |
|  | No | 27,609 | 5,574 | 20.2 | 19.7, 20.7 | 1 |  |  |
|  | Yes | 11,784 | 4,704 | 39.9 | 39.0, 40.8 | 2.63 | 2.51, 2.75 |  |
|  |  |  |  |  | |  | |  |
| Depression, psychological disorders | | |  |  | |  | |  |
| requiring treatment (Q8) | |  |  |  | |  | | <0.001 |
|  | No | 36,334 | 8,639 | 23.8 | 23.3, 24.2 | 1 |  |  |
|  | Yes | 3,059 | 1,639 | 53.6 | 51.8, 55.4 | 3.70 | 3.43, 3.99 |  |
|  |  |  |  |  | |  | |  |
| Sleeping pill use | |  |  |  | |  | |  |
| (> 3 times/week) | |  |  |  | |  | | <0.001 |
|  | No | 35,661 | 8,611 | 24.1 | 23.7, 24.6 | 1 |  |  |
|  | Yes | 3,417 | 1,588 | 46.5 | 44.8, 48.2 | 2.73 | 2.54, 2.93 |  |
|  | MV | 315 | 79 | 25.1 | 20.4, 30.2 | - |  |  |
|  |  |  |  |  | |  | |  |
| Psychotropic drug use | |  |  |  | |  | |  |
| (> 3 times/week) | |  |  |  | |  | | <0.001 |
|  | No | 33,061 | 7,312 | 22.1 | 21.7, 22.6 | 1 |  |  |
|  | Yes | 6,017 | 2,887 | 48.0 | 46.7, 49.3 | 3.25 | 3.07, 3.44 |  |
|  | MV | 315 | 79 | 25.1 | 20.4, 30.2 | - |  |  |
|  |  |  |  |  | |  | |  |
| Depression, anxiety, | |  |  |  | |  | |  |
| tears at menopause | |  |  |  | |  | | <0.001 |
|  | No | 30,807 | 6,821 | 22.1 | 21.7, 22.6 | 1 |  |  |
|  | Yes | 7,930 | 3,338 | 42.1 | 41.0, 43.2 | 2.56 | 2.43, 2.69 |  |
|  | MV | 656 | 119 | 18.1 | 15.3, 21.3 | - |  |  |
|  |  |  |  |  | |  | |  |

Abbreviations: hDS, Presenting high depressive symptoms (CES-D score≥16); MV, Missing Value; N: Number of women.

^a^ Two sided p value for the overall likelihood ratio test
