# Supplementary material for: Dealing with missing data in the Center for Epidemiologic Studies Depression self-report scale: a study based on the French E3N cohort
Source: BMC Med Res Methodol. 2013 Feb 21;13:28. doi: 10.1186/1471-2288-13-28 (PMC3602286; doi:10.1186/1471-2288-13-28)
Supplement: Additional file 6 — Prevalence of high depressive symptoms according to the variables related to morbidities and behavioral characteristics among complete cases (N = 39,393). [file 1471-2288-13-28-S6.doc]

Prevalence of high depressive symptoms according to the variables related to morbidities and behavioral characteristics among complete cases (N=39,393).

|  |  |  |  | Prevalence of high depressive symptoms | | | | |
| --- | --- | --- | --- | --- | --- | --- | --- | --- |
|  |  | N | N hDS | % hDS | 95% CI | OR | 95% CI | *P*^a^ |
|  |  |  |  |  | |  | |  |
| Chronic diseases | |  |  |  | |  | | <0.001 |
|  | 0 | 11,645 | 2,301 | 19.8 | 19.0, 20.5 | 1 |  |  |
|  | 1 | 14,223 | 3,552 | 25.0 | 24.3, 25.7 | 1.35 | 1.27, 1.43 |  |
|  | 2 | 8,442 | 2,545 | 30.1 | 29.2, 31.1 | 1.75 | 1.64, 1.87 |  |
|  | 3 | 3,360 | 1,152 | 34.3 | 32.7, 35.9 | 2.12 | 1.95, 2.31 |  |
|  | 4 or more | 1,723 | 728 | 42.3 | 39.9, 44.6 | 2.97 | 2.67, 3.30 |  |
|  |  |  |  |  | |  | |  |
| Recent hospitalization (<2y) | |  |  |  | |  | | <0.001 |
|  | No | 32,528 | 8,230 | 25.3 | 24.8, 25.8 | 1 |  |  |
|  | Yes | 6,865 | 2,048 | 29.8 | 28.8, 30.9 | 1.26 | 1.19, 1.33 |  |
|  |  |  |  |  | |  | |  |
| Alcohol intake (g/d) | |  |  |  | |  | | <0.001 |
|  | 0 | 4,105 | 1,258 | 30.6 | 29.2, 32.1 | 1 |  |  |
|  | 0 - 2 | 6,100 | 1,721 | 28.2 | 27.1, 29.4 | 0.89 | 0.82, 0.97 |  |
|  | 2 - 4 | 4,329 | 1,115 | 25.8 | 24.5, 27.1 | 0.79 | 0.71, 0.86 |  |
|  | 4 - 8 | 6,311 | 1,535 | 24.3 | 23.3, 25.4 | 0.73 | 0.67, 0.79 |  |
|  | 8 - 16 | 8,556 | 2,022 | 23.6 | 22.7, 24.5 | 0.70 | 0.64, 0.76 |  |
|  | 16 - 32 | 6,955 | 1,752 | 25.2 | 24.2, 26.2 | 0.76 | 0.70, 0.83 |  |
|  | > 32 | 2,644 | 742 | 28.1 | 26.4, 29.8 | 0.88 | 0.79, 0.98 |  |
|  | MV | 393 | 133 | 33.8 | 29.2, 38.8 | - |  |  |
|  |  |  |  |  | |  | |  |
| Smoking | |  |  |  | |  | | <0.001 |
|  | Non smoker | 20,554 | 5,185 | 25.2 | 24.6, 25.8 | 1 |  |  |
|  | Former smoker | 15,812 | 4,171 | 26.4 | 25.7, 27.1 | 1.06 | 1.01, 1.11 |  |
|  | Current smoker | 2,783 | 847 | 30.4 | 28.7, 32.2 | 1.30 | 1.19, 1.41 |  |
|  | MV | 244 | 75 | 30.7 | 25.0, 36.9 | - |  |  |
|  |  |  |  |  | |  | |  |
| Sleep time (/day) | |  |  |  | |  | | <0.001 |
|  | < 6 hours | 960 | 460 | 47.9 | 44.7, 51.1 | 1 |  |  |
|  | 6 - 7 hours | 4,681 | 1,725 | 36.9 | 35.5, 38.3 | 0.63 | 0.55, 0.73 |  |
|  | 7 - 8 hours | 11,647 | 3,074 | 26.4 | 25.6, 27.2 | 0.39 | 0.34, 0.45 |  |
|  | 8 - 9 hours | 14,840 | 3,193 | 21.5 | 20.9, 22.2 | 0.30 | 0.26, 0.34 |  |
|  | 9 - 10 hours | 4,936 | 1,091 | 22.1 | 21.0, 23.3 | 0.31 | 0.27, 0.36 |  |
|  | 10 hours and more | 1,469 | 460 | 31.3 | 28.9, 33.8 | 0.50 | 0.42, 0.59 |  |
|  | MV | 860 | 275 | 32.0 | 28.9, 35.2 | - |  |  |
|  |  |  |  |  | |  | |  |

Abbreviations: hDS, Presenting high depressive symptoms (CES-D score≥16); MV, Missing Value; N: Number of women.

^a^ Two sided p value for the overall likelihood ratio test
