# Supplementary figures and images for: Dealing with missing data in the Center for Epidemiologic Studies Depression self-report scale: a study based on the French E3N cohort
Source: BMC Med Res Methodol. 2013 Feb 21;13:28. doi: 10.1186/1471-2288-13-28 (PMC3602286; doi:10.1186/1471-2288-13-28)

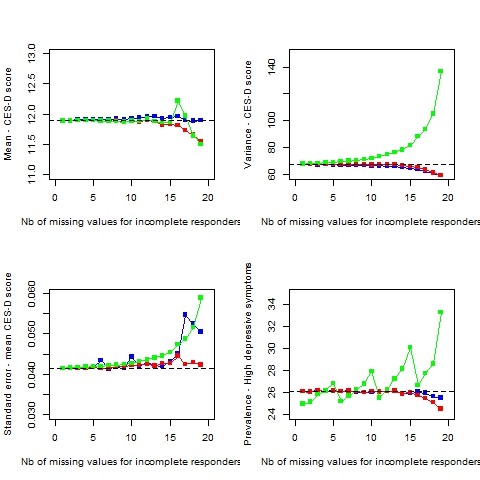

Supplement: Additional file 7 — Results of the simulation study evaluating the predictive accuracy of variouds methods in the case of ignorable MVs. In blue: pmm method – multiple imputation based on a linear regression imputation model. In red: polyreg method – multiple imputation based on a polytomous unordered regression model. In green: single imputation based on the person-mean approach. The dotted lines correspond to the “true” values observed before simulating MVs. [file 1471-2288-13-28-S7.jpeg]
