# Supplementary material for: Dealing with missing data in the Center for Epidemiologic Studies Depression self-report scale: a study based on the French E3N cohort
Source: BMC Med Res Methodol. 2013 Feb 21;13:28. doi: 10.1186/1471-2288-13-28 (PMC3602286; doi:10.1186/1471-2288-13-28)
Supplement: Additional file 8 — Score on the CES-D scale and prevalence of high depressive symptoms after imputation of the values for items considered as quantitative variables, according to various scenarios of nonignorable missing data. [file 1471-2288-13-28-S8.doc]

Score on the CES-D scale and prevalence of high depressive symptoms after imputation of the values for items considered as quantitative variables, according to various scenarios of nonignorable missing data.

|  |  |  | Parsimonious model | | |  | Full model | | |
| --- | --- | --- | --- | --- | --- | --- | --- | --- | --- |
|  |  |  | Score on the CES-D scale | | |  | Score on the CES-D scale | | |
|  |  | N | Mean | SD | ≥ 16 (%) |  | Mean | SD | ≥ 16 (%) |
|  |  |  |  |  |  |  |  |  |  |
| Scenario 1 | | | |  |  |  |  |  |  |
| θ^a^ = 0.1 for all items | | | |  |  |  |  |  |  |
|  |  |  |  |  |  |  |  |  |  |
|  | 0 - 20 MV | 71,412 | 13.58 | 9.14 | 33.22 |  | 13.54 | 9.15 | 32.98 |
|  |  |  |  |  |  |  |  |  |  |
|  | 0 - 10 MV | 62,053 | 12.84 | 8.75 | 30.06 |  | 12.84 | 8.75 | 30.10 |
|  |  |  |  |  |  |  |  |  |  |
|  | 0 - 4 MV | 59,562 | 12.53 | 8.49 | 28.84 |  | 12.53 | 8.50 | 28.85 |
|  |  |  |  |  |  |  |  |  |  |
| Scenario 2 | | | | |  |  |  |  |  |
| θ^a^ = 0.1 for N items, θ^a^ = 0.2 for P items | | | | |  |  |  |  |  |
|  |  |  |  |  |  |  |  |  |  |
|  | 0 - 20 MV | 71,412 | 13.64 | 9.17 | 33.44 |  | 13.60 | 9.18 | 33.24 |
|  |  |  |  |  |  |  |  |  |  |
|  | 0 - 10 MV | 62,053 | 12.86 | 8.76 | 30.14 |  | 12.86 | 8.76 | 30.16 |
|  |  |  |  |  |  |  |  |  |  |
|  | 0 - 4 MV | 59,562 | 12.54 | 8.49 | 28.89 |  | 12.55 | 8.50 | 28.90 |
|  |  |  |  |  |  |  |  |  |  |
| Scenario 3 | | | | |  |  |  |  |  |
| θ^a^ = 0.25 for N items, θ^a^ = 0.5 for P items | | | | |  |  |  |  |  |
|  |  |  |  |  |  |  |  |  |  |
|  | 0 - 20 MV | 71,412 | 14.15 | 9.48 | 35.83 |  | 14.11 | 9.45 | 35.59 |
|  |  |  |  |  |  |  |  |  |  |
|  | 0 - 10 MV | 62,053 | 13.01 | 8.83 | 30.75 |  | 13.01 | 8.82 | 30.75 |
|  |  |  |  |  |  |  |  |  |  |
|  | 0 - 4 MV | 59,562 | 12.64 | 8.52 | 29.30 |  | 12.65 | 8.52 | 29.33 |
|  |  |  |  |  |  |  |  |  |  |
| Scenario 4 | | | |  |  |  |  |  |  |
| θ^a^ = 1 for all items | | | |  |  |  |  |  |  |
|  |  |  |  |  |  |  |  |  |  |
|  | 0 - 20 MV | 71,412 | 16.23 | 11.48 | 41.76 |  | 16.15 | 11.32 | 41.79 |
|  |  |  |  |  |  |  |  |  |  |
|  | 0 - 10 MV | 62,053 | 13.58 | 9.17 | 33.10 |  | 13.56 | 9.10 | 33.14 |
|  |  |  |  |  |  |  |  |  |  |
|  | 0 - 4 MV | 59,562 | 13.03 | 8.63 | 31.00 |  | 13.02 | 8.60 | 31.01 |
|  |  |  |  |  |  |  |  |  |  |

Abbreviations: MV, Missing value; N items, negative items; P items, positive items.

a: Parameter for the MNAR scenario, corresponding to the difference in expected values between subjects with and without MV.
