# Supplementary material for: Dealing with missing data in the Center for Epidemiologic Studies Depression self-report scale: a study based on the French E3N cohort
Source: BMC Med Res Methodol. 2013 Feb 21;13:28. doi: 10.1186/1471-2288-13-28 (PMC3602286; doi:10.1186/1471-2288-13-28)
Supplement: Additional file 9 — Prevalence of high depressive symptoms after imputation of the presenting / not presenting depressive symptoms status, according to various scenarios of nonignorable missing data. [file 1471-2288-13-28-S9.doc]

Prevalence of high depressive symptoms after imputation of the presenting / not presenting depressive symptoms status, according to various scenarios of nonignorable missing data.

|  |  |  | Score on the CES-D scale |
| --- | --- | --- | --- |
|  |  | N | ≥ 16 (%) |
|  |  |  |  |
| Scenario 1 | |  |  |
| θ^a^ = 1.2 | |  |  |
|  |  |  |  |
|  | 0 - 20 MV | 71412 | 31.83 |
|  |  |  |  |
|  | 0 - 10 MV | 62053 | 30.70 |
|  |  |  |  |
|  | 0 - 4 MV | 59562 | 29.55 |
|  |  |  |  |
| Scenario 2 | |  |  |
| θ^a^ = 1.5 | |  |  |
|  |  |  |  |
|  | 0 - 20 MV | 71412 | 32.83 |
|  |  |  |  |
|  | 0 - 10 MV | 62053 | 31.14 |
|  |  |  |  |
|  | 0 - 4 MV | 59562 | 29.92 |
|  |  |  |  |
| Scenario 3 | |  |  |
| θ^a^ = 2.0 | |  |  |
|  |  |  |  |
|  | 0 - 20 MV | 71412 | 34.14 |
|  |  |  |  |
|  | 0 - 10 MV | 62053 | 31.75 |
|  |  |  |  |
|  | 0 - 4 MV | 59562 | 30.37 |
|  |  |  |  |

Abbreviation: MV, Missing value.

a: Parameter for the MNAR scenario, corresponding to the odds ratio expressing the excess risk to present one of the response categories (*i.e.* high depressive symptoms) compared to the reference, in subjects with MV compared to subjects without MV.
